# MicroRNA-146a expresses in interleukin-17 producing T cells in rheumatoid arthritis patients

**DOI:** 10.1186/1471-2474-11-209

**Published:** 2010-09-15

**Authors:** Takuya Niimoto, Tomoyuki Nakasa, Masakazu Ishikawa, Atsushi Okuhara, Bunichiro Izumi, Masataka Deie, Osami Suzuki, Nobuo Adachi, Mitsuo Ochi

**Affiliations:** 1 Department of Orthopaedic Surgery, Programs for Applied Biomedicine, Division of Clinical Medical Science, Graduate School of Biomedical Sciences, 1-2-3, Kasumi, Minami-ku, Hiroshima 734-8551 Japan

## Abstract

**Background:**

Interleukin (IL)-17 is an important factor in rheumatoid arthritis (RA) pathogenesis. MicroRNA (miRNA)s are a family of non coding RNAs and associated with human diseases including RA. The purpose of this study is to identify the miRNAs in the differentiation of IL-17 producing cells, and analyze their expression pattern in the peripheral blood mononuclear cells (PBMC) and synovium from RA patients.

**Methods:**

IL-17 producing cells were expanded from CD4+T cell. MiRNA microarray was performed to identify the miRNAs in the differentiation of IL-17 producing cells. Quantitative polymerase chain reaction was performed to examine the expression patterns of the identified miRNAs in the PBMC and synovium from RA and osteoarthritis (OA) patients. Double staining combining *in situ *hybridization and immunohistochemistry of IL-17 was performed to analyze the expression pattern of identified miRNA in the synovium.

**Results:**

Six miRNAs, let-7a, miR-26, miR-146a/b, miR-150, and miR-155 were significantly up regulated in the IL-17 producing T cells. The expression of miR-146a and IL-17 was higher than in PBMC in the patients with low score of Larsen grade and short disease duration. MiR-146a intensely expressed in RA synovium in comparison to OA. MiR-146a expressed intensely in the synovium with hyperplasia and high expression of IL-17 from the patients with high disease activity. Double staining revealed that miR-146a expressed in IL-17 expressing cells.

**Conclusion:**

These results indicated that miR-146a was associated with IL-17 expression in the PBMC and synovium in RA patients. There is the possibility that miR-146a participates in the IL-17 expression.

## Background

Rheumatoid arthritis (RA) is characterized by chronic synovial inflammation and subsequent joint destruction [1]. The infiltration of macrophages, T cells and B cells in RA synovium plays a crucial role in RA pathogenesis including proliferation of the lining cells, and production of inflammatory cytokines such as tumor necrosis factor- (TNF-)α, and interleukin-1- (IL-1-)β. However, the pathogenesis of RA has not been completely elucidated.

The discovery of a new linage of CD4+ effector T helper type17 cells (Th17cells) that selectively produce IL-17 has provided exciting new insights into immune regulation, host defense, and pathogenesis of autoimmune and other chronic inflammatory disorders including of RA [2-4]. IL-17 is a proinflammatory cytokine, which induces other cytokines, such as TNFα, IL-1β, IL-6, IL-23 and G-CSF [5-8]. In addition, IL-17 plays a role in osteoclastogenesis via activation of RANKL(receptor activator of NF-κB ligand), causing bone destruction in inflammatory joints [9-11]. Several studies demonstrated that IL-17 is higher in synovial fluid, synovium and peripheral blood mononuclear cells in RA patients than that in healthy subjects [12-14]. IL-17 is recognized to be the one of the important factors in RA pathogenesis.

MicroRNA (miRNA)s are a family of ~22-nucleotide non coding RNAs identified in organisms ranging from nematodes to humans [15-17]. Many miRNAs are evolutionarily conserved across phyla, thereby regulating gene expression by posttranscriptional gene repression. The miRNAs regulate gene expression by binding the 3'-untranslated region of their target mRNAs leading to translational repression or mRNA degradation [18-21]. Several microRNAs exhibit a tissue-specific or developmental stage-specific expression pattern and have been reported to be associated with human diseases such as cancer, leukemia, and viral infection [22,23]. These findings suggest their potential as a novel therapeutic target. miRNA might play a role in RA pathogenesis in autoimmune and other chronic inflammatory diseases including of RA. Several studies reported that miRNA might play a role in RA pathogenesis. Stancyzk et al. reported that miR-146 and miR-155 are highly expressed in RA synovial fibroblast in comparison to osteoarthritis fibroblast [24]. Nakasa et al. demonstrated that miR-146 is highly expressed in RA synovial tissue in comparison to OA and normal synovial tissue. They also revealed that miR-146 is expressed primarily in CD68+ macrophages, but also in some CD3+ T cell subsets and CD79a+ B cells in RA synovial tissue [25]. Paulay et al. reported that PBMCs from RA patients exhibit statistically significant increase the expression levels of miR-146a, miR-155, miR-132, and miR-16 in comparison to healthy and disease control individuals. They also demonstrated that high levels of miR-146a and miR-16 expression correlate with active disease, whereas low expression levels correlate with inactive disease [26]. Changsheng Du et al. reported that miR-326 regulates Th-17 differentiation and associated with the pathogenesis of multiple sclerosis[27]. These findings suggest that miRNA also might play a role in the expression of IL-17, and an analysis of the expression pattern of miRNAs in IL-17 producing T cells might lead to the development of new treatments for RA.

The purpose of this study is to identify miRNAs in the differentiation of IL -17 producing T cells, and to analyze their expression pattern in the RA patients. MiRNAs were identified during the differentiation of IL-17 producing T cells by the expansion from healthy CD4+ T cells using microarray analysis, and analyzed the expression pattern of the identified miRNAs in the peripheral blood mononuclear cells (PBMC) and synovium from RA and OA patients.

## Methods

### Patients

This clinical study was approved by the University of Hiroshima Institutional Review Board, and written permission was obtained from all patients who participated in this study. Peripheral blood was taken from 6 RA patients (67.3 ± 8.02 years of age, mean ± standard deviation (SD)) and 6 OA patients (75.3 ± 5.0 years of age, mean ± SD). They were diagnosed according to the American Rheumatism Association Criteria for RA or OA[28]. Synovial tissue specimens were obtained at the time of total knee arthroplasty on 4 RA patients and two RA patients (RA8,RA9) were obtained at the time of synovectomy (67.7 ± 10.4 years of age, mean ± SD). All OA synovial tissue samples were obtained by total knee arthroplasty (74 ± 5.1 years of age, mean ± SD). Patient RA3 and RA5 showed more erosive disease, with severe destruction in the large joints. They underwent total knee arthroplasty before, and they were subsequently well controlled. The disease in patients RA1, RA2 and RA4 were poorly controlled, but soft tissue swelling, juxta-articular osteopenia, and loss of joint space were observed. Patient RA6, RA7, RA10 and RA11 showed more erosive disease, with severe destruction in the large joints. RA8 and RA9 had the least erosive disease. In addition, 6 patients with knee osteoarthritis (OA) diagnosed according to typical clinical features. The demographics of the RA and OA patients are listed in Table 1.

**Table 1:** Demographic and clinical features of this study subjects.

			*disease duration Larsen grade K/L score*			*CRP*	*ESR*		*synovium*		
*subject*	*age*	*sex*						*RF*	*PBMC*		*medication*
			*(yr)*	*for RA*	*for OA*	*(mg/ml)*	*(mm/hr)*		*(source)*		
RA1	76	F	4	Ⅰ		0.53	45	209.3	○		MTX, NSAIDs
RA2	69	M	3	Ⅱ		0.31	52	22.9	○		bucillamine, NSAIDs
RA3	59	M	16	Ⅴ		3.53	no data	49.5	○		MTX, predonisone, NSAIDs
RA4	59	F	2	Ⅰ		4.12	74	129.2	○		salazosulfapyridine
RA5	64	M	17	Ⅴ		2.57	48	65.3	○		bucillamine, NSAIDs
RA6	77	F	3	Ⅳ		0.16	66	160.9	○	○ (knee)	salazosulfapyridine
RA7	63	F	14	Ⅳ		0.33	21	<6.0		○ (knee)	infliximab
RA8	54	M	3	Ⅲ		3.07	no data	no data		○ (elbow)	MTX, predonisone, NSAIDs
RA9	79	M	5	Ⅲ		0.03	8	6.1		○ (wrist)	NSAIDs
RA10	74	F	15	Ⅳ		0.33	27	no data		○ (knee)	bucillamine, NSAIDs
RA11	59	F	5	Ⅳ		3.55	61	60.8		○ (knee)	MTX, bucillamine, predonisone, NSAIDs
											
OA1	74	F			Ⅳ				○		NSAIDs
OA2	78	F			Ⅳ				○	○ (knee)	NSAIDs
OA3	83	F			Ⅳ				○	○ (knee)	NSAIDs
OA4	71	F			Ⅲ					○ (knee)	NSAIDs
OA5	76	M			Ⅳ				○		NSAIDs
OA6	70	F			Ⅳ				○		NSAIDs
OA7	71	F			Ⅳ				○		NSAIDs
OA8	74	F			Ⅳ					○ (knee)	NSAIDs
OA9	70	F			Ⅳ					○ (knee)	NSAIDs
OA10	71	F			Ⅳ					○ (knee)	NSAIDs

RA : rheumatoid arthritis; OA : osteoarthritis; K/L : Kellgren/Lawrence; CRP : C-reactive protein; ESR : erythrocyte sediment rate; RF : rheumatoid factor; MTX : methotrexate; NSAIDs : nonsteroidal anti-inflammatory drugs;

### Cell isolation and expansion of IL-17 producing T cells

This clinical study was approved by the University of Hiroshima Institutional Review Board, and written permission was obtained from all healthy volunteers who participated in this study. Human peripheral blood was collected from 5 healthy volunteers (31.8 ± 1.1 years of age, mean ± SD) into DPBS-E (5 mM: 0.5 M EDTA) drop by drop and mixed well. This mixture was loaded slowly onto Histopaque^® ^(Sigma Chemical Co. CA) in another tube, and centrifuged at 1000 × g at room temperature for 10 min. PBMC accumulated as the middle white monolayer. After the supernatant was discarded, only the white monolayer cells were aspirated and put into DPBS-E, then centrifuged at 400 × g at 4°C for 10 min. The supernatant was discarded, DPBS-E and ammonium chloride (The cell Experts™) were added at the rate of 1:3, and the mixture was allowed to stand at room temperature for 10 min. After centrifuging at 400 × g at 4°C for 10 min, supernatant was discarded, and DPBS-E was added and mixed well. This final process was repeated several times. The remaining white cells were peripheral blood mononuclear cells (PBMC). CD4+ T cells were isolated from PBMC using *auto MACS *(CD4+T cell Isolation kit, Miltenyi Biotec,). IL-17 producing cells were expanded from CD4+ T cells as previously described [29]. PBMC or purified CD4+ T cells (1 × 10^6 ^per ml) were re-suspend in fresh culture medium containing requisite antibiotics and serum plus IL-2 (100 u/ml), IL -1 β, IL-2, IL -6 and IL -23 (R&D Systems; 10ng/ml). The cells were cultured in 24 well plates (2 ml per well) at 37°C for 4 days in 5% CO_2_. Afterwards, IL-17 expression was confirmed with RT-PCR and ELISA, and the presence of IL-17 producing T cells was also confirmed (data is not shown).

### Tissue samples

Three synovial tissue specimens per one patient were obtained from random sites during surgery. Each was visually inspected to minimize contamination with non-inflammatory tissue. Tissues were stored at -70°C until analysis. Total RNA for the PCR analysis was isolated from tissues homogenized with Trizol (Invitrogen) on ice for. The tissue specimens were fixed in 4% paraformaldehyde and paraffin-embedded for histopathological analysis.

### Microarray analysis

miRNA microarrays (NCode microarray, Invitrogen) were performed for the identification of miRNA in differentiating IL-17 producing cells. The dual-color dye swap method was used to analyze differences of miRNA expression between expanded IL-17 producing cells and non-expanded cells. Five-hundred nanograms of the enriched miRNA was labeled with the NCode Rapid miRNA labeling kit (Invitrogen) and hybridized to NCode multispecies miRNA arrays as described earlier based on a loop design, balanced with respect to array, dye, and sample. The balanced design minimized sources of unwanted variation. The arrays were scanned, aligned, and median spot intensities were obtained using a GenePix 4000B scanner (Molecular Devices, Inc., Sunnyvale, CA, USA).

### Synthesis of complementary DNA

One microgram of total RNA was reverse-transcribed using the QuantiTect^® ^Reverse Transcription Kit (Qiagen, Chatsworth, CA) according to the manufacturer's protocol. The genomic DNA elimination reaction was carried out using 2 μl of gDNA wipeout buffer, 1 μg (1 μl) template RNA and 11 μl RNase-free water at 42°C for 2 min. Reverse transcription was performed in 1 μl quantiscript reverse transcriptase, 4 μl quantiscript RT buffer, 1 μl RT primer mix and 14 μl template RNA (the entire genomic DNA elimination reaction) at 42°C for 15 min and 95°C for 3 min and then the cDNA product was maintained at 4°C.

### Quantitative (real time) PCR

Quantitative RT-PCR assays were performed using a TaqMan miRNA assay kit (Applied Biosystems, CA, USA) for the expression of miRNAs and SYBR Green (Invitrogen) for the expression of IL-17, Foxp3, retinoid-related orphan receptor γt (RORγt), IL-1 receptor-associated kinase 1(IRAK1) and suppressor of cytokine signaling 1(SOCS1). Reverse transcriptase reactions of mature miRNAs contained a sample of total RNA, 50 nM stem-loop RT primer, 10 × RT buffer, 100 mM each dNTPs, 50 U/μl MultiScribe reverse transcriptase, and 20 U/μl RNase inhibitor. 15 μl reactions were incubated in a thermo cycler (BioRad) for 30 min at 16°C, 30 min at 42°C, 5 min at 85°C, and held at 4°C. Real time PCR was performed using a Mini Opticon Real-time PCR System (BioRad, Hercules, CA) in a 10 μl PCR mixture containing 1.33 μl RT product, 2 × TaqMan Universal PCR Master Mix, 0.2 μM TaqMan probe, 15 μM forward primer, and 0.7 μM reverse primer. Each SYBR Green reaction was performed with 1.0 μl template cDNA, 10 μl SYBR Green mix, 1.5 μM primer, and water to adjust the final volume to 20 μl. Primer sequences were: IL-17, 5'- AAG ACC TCA TTG GTG TCA CTG CT AC-3'(forward), 5'- ATC TCT CAG GGT CCT CAT TGC G-3' (reverse); Foxp3, 5'- GAG AAG CTG AGT GCC ATG CA -3'(forward), 5'- AGA GCC CTT GTC GGA TGA T -3'(reverse); RORγt, 5'- TGA GAA GGA CAG GGA GCC AA-3'(forward), 5'- CCA CAG ATT TTG CAA GGG ATC A -3' (reverse); SOCS1, 5'- GAA CTG CTT TTT CGC CCT TA -3'(forward), 5'- CTC GAA GAG GCA GTC GAA G -3'(reverse); IRAK1, 5'- GCT CTT TGC CCA TCT CTT TG -3'(forward), 5'- GCT ACC ACG CCA GGC TAA TA -3'(reverse); GAPDH, 5'- AAG AAT TGC AAG TCT ACA TAT CAC CCA AG. -3'(forward), 5'- GGT CAT GGT CAC AGA GCC ACC-3'(reverse). All reactions were incubated in a 48 well plate at 95°C for 10 min, followed by 40 cycles of 95°C for 15 seconds, and 60°C for 1 min and performed in triplicate. The U18 or GAPDH gene was used as a control to normalize any differences in the total RNA levels in each sample. A threshold cycle (C_T_) was observed in the exponential phase of amplification, and quantification of relative expression levels was performed using standard curves for target genes and the endogenous control. Geometric means were used to calculate the ΔΔC_T _(delta-delta C_T_) values and expressed as 2^-ΔΔCT^. The value of each control sample was set at 1 and was used to calculate the fold-change of target genes.

### Western Blotting

Ten μg of the protein were separated on NuPAGE^® ^Novex^® ^Bis-Tris Mini Gels (Invitrogen, Carlsband, CA) and transferred onto a nitrocellulose membrane (Invitrogen, Carlsband, CA). Mouse monoclonal antibody against a partial recombinant IRAK1 (Abnova, Taiwan) and rabbit polyclonal anti-actin antibody (Santa Cruz Biotechnology, Santa Cruz, CA) were used as primary antibodies. Anti-mouse goat IgG (MP Biomedicals, LLC, Santa Ana, CA) for IRAK1 and anti-rabbit goat IgG (MP Biomedicals, LLC, Santa Ana, CA) for actin were used for secondary antibodies. Band detection was performed using the enhanced chemiluminescence reagent, ECL Western Blotting Detection Reagents (GE Healthcare UK Ltd, Little Chalfont, Buckinghamshire).

### Immunohistochemistry

Paraffin sections were deparaffinized through xylene for three changes of five minutes each, followed by graded alcohol immersions to water and phosphate buffered saline solutions, the sections were treated with the retrieval solutions (DAKO, Dakocytomation Inc.,Copinteria,CA,USA) for 20 minutes at 95°C. Next, the sections were depleted of endogenous peroxidase by incubation with 0.3% H_2_O_2 _in absolute methanol for 15 minutes. After blocking nonspecific biding with blocking reagent for 30 minutes, the sections incubated with primary antibody at appropriate dilutions for overnight at 4°C. For primary antibodies, monoclonal rabbit anti-human antibody against IL-17 (Santa Cruz Biotechnology,Inc,CA,USA). The sections were washed and incubated with biotinylated goat anti-mouse (Sigma, Saint Louis, Missouri, USA) for 1 hour at room temperature, then, washed and incubated with avidin-biotinylated horseradish peroxidase complex (ABC) and diaminobenzidine tetrahydrochloride (DAB; Dakocytomation Inc.,Copinteria,CA), and counterstained with Mayer's hematoxylin. The negative control was prepared in the same manner except that the primary antibody was omitted.

### Double staining combining in situ hybridization and immunohistochemistry

After deparaffinization, each section was fixed in 4% paraformaldehyde for 10 minutes at room temperature, washed 3 times in phosphate buffered saline (PBS) for 3 minutes, and subsequently treated with 600 g of proteinase K for 10 minutes at room temperature. After treatment in 0.2% glycine-PBS for 10 minutes, the sections were refixed in 4% paraformaldehyde for 10 minutes, washed 3 times in PBS for 3 minutes each, and acetylated with 0.25% acetic anhydride in 0.1M triethanolamine hydrochloride for 10 minutes. After washing in PBS for 30 minutes, sections were prehybridized for 1 hour at 65°C with prehybridization buffer (50% formamide and 5 saline-sodium citrate [SSC]). Hybridization with DIG-labeled riboprobes of miR-146a (B-Bridge International, Mountain View, CA) was performed overnight　at 65°C in hybridization buffer (50% formamide, 5X SSC, 5X Denhardt's solution, and 250 g/ml of Baker's yeast transfer RNA). After hybridization, sections were washed in 5X SSC for 30 minutes at 65°C, 0.2 SSC for 2 hours at 65°C, and 0.2X SSC for 5 minutes at room temperature. Blocking was performed overnight at 4°C with 4% horse serum and alkaline phosphatase-conjugated Fab anti-DIG antibody (Roche) in 1% sheep serum. Staining was performed using BCIP and nitroblue tetrazolium (NBT; Roche). Thereafter, the sections were washed in PBS and then were treated for 20 minutes at 90°C with retrieval solutions (Dakocytomation Inc.,Copinteria,CA). After blocking for 30 minutes, the sections were incubated with primary antibody of IL-17 at appropriate dilutions for 1 hour at room temperature. After washing, the sections were incubated with Alexa Fluor 594 conjugate(Invitrogen, Carlsbad, CA) for 30 minutes at room temperature, washed, and then incubated with 4, 6-diamidino-2-phenylindole (Dojindo Laboratories, Kumamoto, Japan). The negative control was prepared in the same manner, but without the primary antibody.

### Statistical analysis

The Mann-Whitney U test was used to compare the gene expression between two groups. A one-way analysis of variance (ANOVA) followed by Tukey's post hoc analysis was used to compare gene expression between the three groups. P values less than 0.05 were considered to be statistically significant. All statistical analyses were performed on a personal computer using the Stat View version 5.0 statistical software package (Abacus Concepts, Berkeley, CA).

## Results

### Six miRNAs up-regulated in IL-17 producing T cells in microarray analysis

**Figure 1:**
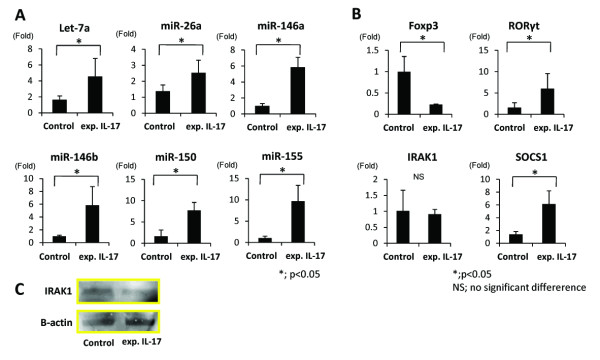
**A quantitative PCR analysis of the expression of six miRNAs, Foxp3, RORγt, IRAK1, and SOCS1**. A, A quantitative PCR analysis of the expression of let-7a, miR-26a, 146a,b, 150, and 155 in expanded IL-17 producing T cells and non-expanded cells. The expression level of these six miRNAs in expanded IL-17 producing T cells was significantly higher than in non-expanded cells. MiR-146a and miR-146b have increased by a factor of six in comparison to non-expanded cells. miR-155 showed a 9-fold increase, and miR-26a showed the smallest increase. B, A quantitative PCR analysis of the expression of Foxp3, RORγt, IRAK1, SOCS1. The expression level of RORγt, and SOCS1 in expanded IL-17 producing T cells significantly was higher than in non-expanded cells, while Foxp3 in expanded cells was significantly down regulated in comparison to non expanded cells. There was no significant difference in the expression of IRAK1. C,Western blotting of IRAK1. IRAK1 was down regulated in expanded IL-17 producing T cell in protein level.

The microarray analysis of the expanded IL-17 producing T cells identified six miRNAs that were significantly up regulated during the differentiation of the IL-17 producing T cells(The Center for Information Biology Gene Expression Database, CIBEX; http://cibex.nig.ac.jp. CIBEX Accession : CBX133.). The six miRNAs were miR-26a (1.81 fold; p = 0.029), 146a (1.43 fold; p = 0.040), 146b (1.80 fold; p = 0.040), 150 (1.61 fold; p = 0.040), 155 (2.03 fold; p = 0.007) and let-7a (2.12 fold; p = 0.012; Table 2). There was no miRNA that was significantly down regulated in the differentiation of IL-17 producing T cells. The expression of let-7a, miR-26a, 146a,b, 150, and 155 between expanded IL-17 producing T cells and non-expanded cells was compared by using real-time PCR to confirm the results of microarray analysis. The expression level of these six miRNAs in expanded IL-17 producing T cells was significantly higher than in CD4+ T cells. MiR-146a and miR-146b increased by a factor of six in comparison to non-expanded cells. The expansion of miR-155 was the greatest (9-fold), and the lowest was miR-26a (Figure 1A). Real-time PCR was performed to examine the expression of the important factors in the differentiation of human Th17 cells, including Foxp3, RORγt, IRAK1, and SOCS1. The expression level of RORγt, and SOCS1 in expanded IL-17 producing T cells was significantly higher than in non-expanded cells, while Foxp3 in CD4+ T cells was strongly expressed than in expanded IL-17 producing T cells. There was no significant difference in the expression of IRAK1between CD4+ T cells and expanded IL-17 producing T cells (Figure 1B). However, western blotting revealed that IRAK1 was down regulated in expanded IL-17 producing T cells in protein level (Figure 1C).

**Table 2:** Six miRNAs up-regulated in IL-17 producing T cells in microarray analysis.

	miRNA ID	Expression ratio	p value
**6 miRNAs up-regulated**	let7a	2.119413042	0.01219878
	hsa-miR-26a	1.812510994	0.02939706
	hsa-miR-146a	1.43106906	0.04019598
	hsa-miR-146b	1.802918862	0.03959604
	hsa-miR-150	1.605962505	0.04019598
	hsa-miR-155	2.025291111	0.00719928

For the identification of miRNA in differentiating IL-17 producing cells, miRNA microarrays (NCode microarray, Invitrogen) were performed. Six miRNAs were significantly up regulated in the differentiation of the IL-17 producing T cells. There was no miRNA that significantly down regulated in the differentiation of IL-17 producing T cells. (The Center for Information Biology Gene Expression Database, CIBEX; http://cibex.nig.ac.jp. CIBEX Accession : CBX133.)

### RA patient PBMCs exhibit increased expression of Let-7a, miR-26a, 146a,b, 150, and 155

**Figure 2:**
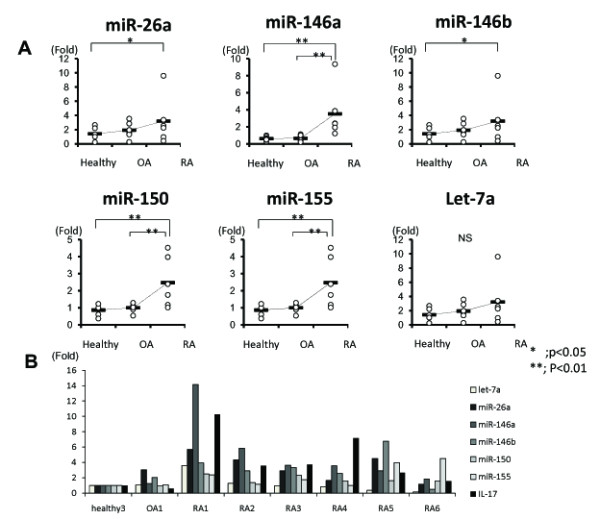
**A quantitative PCR analysis for the expression of six miRNAs in PBMC**. A, A quantitative PCR analysis for the expression of six miRNAs in PBMC in RA, OA patients and healthy volunteer. The expression level of miR-26a, miR-146a/b, miR-150 and miR-155 in RA patients were significantly higher than in healthy subjects. There were no significant differences between RA and OA patients in the expression level of miR-26a, miR-146b. Between RA and OA patients, significant differences were observed in the expression level of miR-146a, miR-150 and miR-155. B,. B., quantitative PCR analysis to examine the relationship between the expression level of Let-7a, miR-26a, 146a/b, 150, and 155 and IL-17 in RA patients. IL-17 and miR-146a was more intensely expressed in patient RA1, RA2 and RA4 than patient RA3,RA5,RA6 and OA1.

The expression pattern of the six miRNAs in PBMC form RA, OA and healthy subjects were analyzed using real-time PCR to determine whether the miRNA identified by microarrays analysis were expressed in RA patients in comparison to OA and healthy subjects,. The expression level of miR-26a, miR-146a/b, miR-150 and miR-155 in RA patients were significantly higher than in healthy subjects. However, there were no significant differences between RA and OA patients in the expression level of miR-26a, miR-146b. There were significant differences between RA and OA patients in the expression level of miR-146a, miR-150 and miR-155. There was no significant difference in the expression level of Let-7a between RA, OA patients and healthy subjects (Figure 2A). Real time PCR of IL-17 was used to examine the relationship between the expression level of six miRNAs and IL-17 in RA patients. The expression level of miR-146a was similar to that of IL-17. Patient RA1 and RA4 had high disease activity, although the Larsen grade was low. The expression level of miR-146a and IL-17 in patient RA1 and RA4 was high in comparison to the other patients. miR-146a expressed intensely with IL-17 expression in PBMC from the patients with early stage of RA and high disease activity (Figure 2B).

### The synovium from RA patients exhibit increased expression of miR-146a,b, 150, and 155

**Figure 3:**
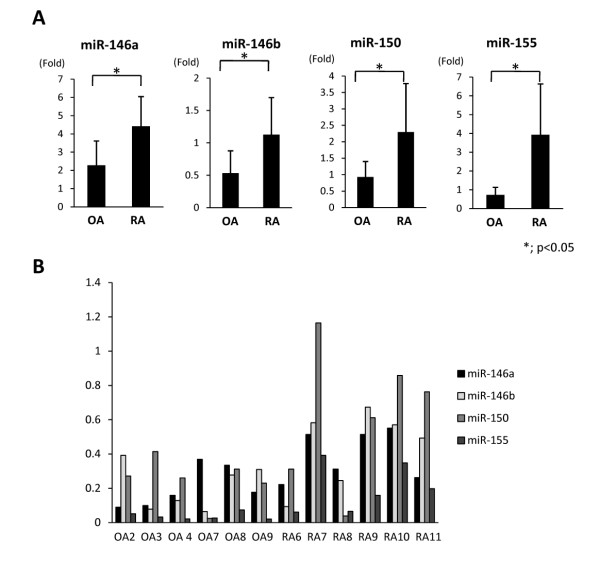
**A quantitative PCR analysis for the expression of miRNAs in synovium**. A, A quantitative PCR analysis for the expression level of miR-146a/b, miR-150, and miR-155 in synovium of RA and OA patients. All miRNAs were intensely expressed in RA synovium in comparison to OA. B, miR-146a and miR-146b tended to be express intensely in high disease activity. miR-150 was highly up regulated in synovium in patients RA7, RA10 and RA11.

**Figure 4:**
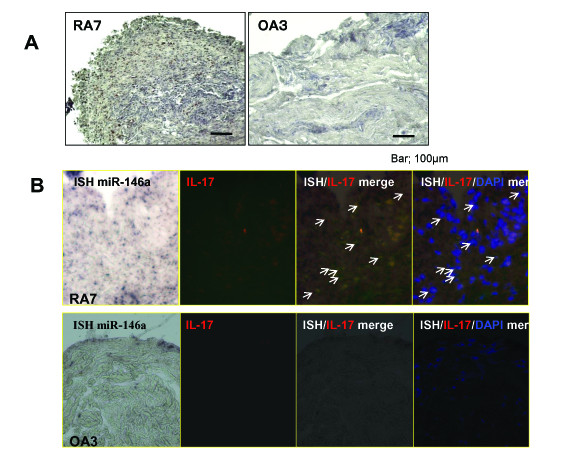
**Immunohistochemistry and double staining combining in situ hybridization**. A, Representative histology of RA and OA by immunohistochemistry of IL-17. RA synovium with high expression level of miR-146a/b, miR-150 and miR-155 demonstrated amount IL-17 positive cells in hyperplasia synovium while OA synovium had few IL-17 positive cells in the fibrous tissue. Original magnification 100×. Bar indicates 100 μm. B, Double staining for the *in situ *hybridization and immunohistochemistry of rheumatoid arthritis (RA) and osteoarthritis synovial tissue. *In situ *hybridization (ISH) for mature microRNA146-a (miR-146a) and immunohistochemistry with IL-17 antibodies were performed on synovial tissue specimens obtained from patient RA7 and OA3. Mature miR-146a was expressed in the cells of the superficial and sublining layers, thus including IL-17 positive cells. The arrows in the merged images indicate the cells expressing miR-146a and IL-17 antibody. Staining of the tissue sections with 4, 6-diamidino-2- phenylindole (DAPI) is shown at the right. (Original magnification X200).

RA pathogenesis is characterized by synovial inflammation, secretion of inflammatory cytokines including IL-17 and subsequent joint destruction. Therefore, the expression pattern of miR-146a/b, miR-150, and miR-155 in the synovium of RA patients was examined using real time PCR. miR-146a, miR-146b, miR-150, and miR-155 were expressed intensely in RA in comparison to OA. There was a significant difference between RA and OA. The expression of miR-146a and miR-146b tended to increase with high disease activity, which was consistent with previous reports[24,25]. In particular, miR-150 was highly up regulated in the synovium of patients RA7, RA10 and RA11, (Figure 3A). Patient RA7 and RA11 were poor controlled with severe joint destruction. Patient RA10 had long disease duration subsequently severe joint destruction (Figure 3B). The disease activity in the synovium was analyzed histologically using IL-17 immunohistochemistry. The synovium with high expression of miR-146a, miR-146b, miR-150 and miR-155 showed vigorous proliferation of synovial cells and infiltration of inflammatory cells and abundant IL-17 positive cells. The analysis of the synovium from RA and OA patients revealed that miR-146a/b, miR-150 and miR-155 were highly expressed in RA synovium with hyperplasia and infiltration of inflammatory cells including Th17 producing cells in poorly controlled patients with severe joint destruction (Figure 4A).

### MiR-146a expressed in IL-17 producing cells

MiR-146a was previuosly reported to be expressed in the T cells in the RA synovium [25]. We therefore confirmed whether miR-146a expressed in the IL-17 producing T cells using the double staining of *in situ *hybridization and immunohistochemistry of IL-17. MiR-146a expression was mainly observed in the superficial and sublining layers as previuosly described [25]. In addition, IL-17 was seen in the RA synovium, while it was not seed in OA synovium. Double staining revealed that miR-146 + cells merged IL-17, thus indicating miR-146a to be expressed in IL-17 producing T cells (Figure 4B).

## Discussion

Recently, a potential link between miRNA and several human diseases has been revealed. For example, the expression of let-7 has been shown to be lower in lung cancer tissue than in normal lung tissue, and such down-regulation may promote high levels of expression of the Ras gene [30]. In addition, the expression of miR-143 and miR-145 is reduced in colon cancer tissue. Evidence of miRNA function in conditions such as leukemia, viral infection, and DiGeorge syndrome has been reported [31,32]. Recent reports have suggested that several miRNA might participate in the pathogenesis of RA [21,24-26].

miRNAs have been investigated because of their potential clinical application for therapeutic methods. Therapeutic trials aimed at silencing miRNA *in vivo *have been conducted [32,33]. Tazawa et al. demonstrated that miR-34a is down-regulated in human colon cancer, and that tumor growth in mice is significantly inhibited by the local injection of synthetic double stranded miR-34a *in vivo *[34]. The intra-articular injection of double stranded miR-15a successfully induced cell apoptosis by inhibiting the translation of BCL2 protein in the synovium in arthritic mice. This suggested that the intra-articular injection of synthetic miRNAs can regulate the endogenous miRNAs in arthritic synovia [35]. The discovery of a new lineage of CD4+ effecter T helper (Th) cells that selectively produce IL-17 in mice has provided exciting new insights into immune regulation, host defense, and the pathogenesis of autoimmune and other chronic inflammatory disorders. Although IL-17 plays a crucial role in RA pathogenesis, the differentiation mechanism of human Th17 cells from CD4+ T cells is unclear. MiRNAs are reported to be a important factor in the differentiation of T cells [36]. Therefore, identification of miRNAs in Th17 cell differentiation could elucidate a new mechanism of RA pathogenesis, subsequently lead a novel therapeutic approach to the RA. In the current study, IL-17 producing T cells were expanded from PBMCs from healthy volunteers and the expression pattern of miRNAs in the differentiation of IL-17 producing cells was analyzed by using a Microarray analysis. This demonstrated that let-7a, miR-26a, 146a,b, 150, and 155 were significantly upregulated in the differentiation of IL-17 producing cells. There are several reports about Foxp3, RORγt, IRAK1 and SOCS1in the differentiation of IL-17 producing T cells. Foxp3 is the transcription factor controlling regulatory T cell development[37], while, RORγt is the master regulator that directs the differentiation program of Th17 cells [38]. The expression level of RORγt in expanded IL-17 producing T cells was significantly higher than in non-expanded cells, while Foxp3 was expressed more strongly in non-expanded cells in comparison to expanded cells, indicating that the IL-17 producing T cells were expanded. IRAK1 and SOCS1 not only play a crucial role in Th17 cell differentiation, but also are regulated by miR-146a/b and miR-155 respectively [39,40]. In the current series, the expression level of SOCS1 in expanded IL-17 producing T cells was significantly higher than in non-expanded cells. There was no significant difference in the expression level of IRAK1 between the expanded and non expanded cells at the mRNA level. However IRAK1 protein was decreased in the expanded cells, which suggested miR-146a/b might inhibit the translation to IRAK1 protein from mRNA.

MiR-146a was expressed intensely with IL-17 expression in PBMC from the patients with early stage of RA and high disease activity. miR-146a/b was associated with high disease activity, and miR-150 was intensely expressed in the patients with severe joint destruction. MiR-150 has not been associated with the high expression of miR-146 and miR-155 in PBMC and synovium of RA in previous reports [24-26]. MiR-150 might also play a role in RA pathogenesis. The expression of miR-146a and IL-17 was high in PBMC in patients with low score of Larsen grade and short disease duration in the current series. A patient with a high score of Larsen grade and long disease duration showed a low expression level of miR-146a, while that of miR-150 was high. Therefore, the high expression of miR-146a in PBMC was strongly associated with IL-17 expression, especially at the early stage of RA. Severe joint destruction with high disease activity was associated with the expression of miR-150 in the synovium. The number of patients in the current study was small, therefore, further examination is necessary to clarify the relationship between these miRNAs and disease activity, including the expression level of IL-17. Our results revealed miR-146a to be expressed in the IL-17 producing T cells. In a previous report, accumulated CD3+ T cells were observed to express miR-146, thereby suggesting that miR-146 might play a role in the persistent inflammation in RA via a T cell network, which strongly supports that miR-146a thus expressed in Th17 cells [25].

The current results suggested that miR-146a/b, miR-150 and miR-155 might play a role in IL-17 producing T cells differentiation and these miRNAs were expressed intensely in the PBMC and synovium in RA patients in comparison to OA patients and healthy subjects. In addition, the miR-146a expression in the IL-17 producing T cells of the RA synovium was confirmed. In the RA patients there is no report that analyzes miRNAs associated with IL-17 producing T cells. Changsheng Du et al. reported that miR-326 regulates Th-17 differentiation and associated with the pathogenesis of multiple sclerosis[27]. These evidence suggested that the function and analysis on the differentiation of IL-17 producing T cells is not simple, and perhaps they were depending on the disease variety, disease progression degree and the disease stage. Indeed, previous reports demonstrated that miR-146a/b, miR-150 and miR-155 are expressed in PBMC or synovium in RA patients, which might include miRNAs which are expressed in IL-17 producing T cells. However, the function of these miRNAs in the differentiation of IL-17 producing T cells was not elucidated in the present study. Several target genes of these 6 miRNAs were validated[30,40-53] (Table 3). The predicted target genes for miRNAs are estimated to range between one and several hundred, therefore, other target genes might play a role in the differentiation of IL-17 producing cells as well as the validated target genes. Further examination is needed to elucidate the function of these miRNAs including the determination of their target genes, which could lead to the development of novel therapeutic strategies for the treatment RA.

**Table 3:** Validated target genes of 6 miRNAs.

miRNA	Targets	Referances
let7a	Lin-41, Hbl-1, RAS, TRIM71	[41], [42], [43], [30], [44]
miR-26a	Ezh2, GSK-3β、	[45], [46]
miR-146a/b	TRAF6, IRAK1,IRAK2	[47], [48]
miR-150	c-Myb	[49]
miR-155	FADD, IKK, Ripk1, TAB2, PU.1, SOCS1, AID	[50], [51], [52], [40], [53]

TRIM71: tripartite motif-containing 71;Ezh2: Enhancer of Zeste homolong2; GSK-3β: glycogen synthase kinase-3β; TRAF6: tumor necrosis factor receptor-associated factor 6; IRAK-1: Interleukin-1 receptor-associated kinase 1; IRAK-2: Interleukin-1 receptor-associated kinase 2;FADD: Fas-associated death domain protein; IKK: IκB kinase; Ripk1: receptor interacting serine-threonine kinase 1; SOCS-1: suppressor of cytokine signaling 1; AID: activation-induced cytidine deaminase;

## Conclusions

These results indicated that miR-146a/b, miR-150 and miR-155 were associated with IL-17 expression in the PBMC and synovium in the RA patients, especially expression of miR-146a in IL-17 producing cells was comfirmed. Their altered expression in the stage and activity of RA suggested that they might play a role in the pathogenesis of RA via IL-17 expression. In addition, our results should be developed the excessive analysis of the function of these miRNAs in the RA to lead the novel treatment.

## Abbreviations

RA: rheumatoid arthritis; OA: osteoarthritis; K/L: Kellgren/Lawrence; CRP: C-reactive protein; ESR: erythrocyte sediment rate; RF: rheumatoid factor; MTX: methotrexate; NSAIDs: nonsteroidal anti-inflammatory drugs; RORγ: retinoid-related orphan receptor γ; SOCS: suppressor of cytokine signaling; IRAK1: IL-1 receptor-associated kinase 1.

## Competing interests

The authors declare that they have no competing interests.

## Authors' contributions

TN and TN designed this study. TN conducted all experiments and drafted manuscript. MD, OA and OS collected patients' samples. NA and MI provided clinical insights and edited manuscript. TN assisted with statistical evaluations. MO conceived of the study, assisted in designing the study, and edited the manuscript. All authors read and approved the final manuscript.

## Pre-publication history

The pre-publication history for this paper can be accessed here:

http://www.biomedcentral.com/1471-2474/11/209/prepub
